# Preparing Size-Controlled Liposomes Modified with Polysaccharide Derivatives for pH-Responsive Drug Delivery Applications

**DOI:** 10.3390/life13112158

**Published:** 2023-11-03

**Authors:** Shin Yanagihara, Yukiya Kitayama, Eiji Yuba, Atsushi Harada

**Affiliations:** 1 Department of Applied Chemistry, Graduate School of Engineering, Osaka Prefecture University, 1-1 Gakuen-cho, Naka-ku, Sakai 599-8531, Osaka, Japan; liushin.ynghr@gmail.com (S.Y.); kitayama@omu.ac.jp (Y.K.); atsushi_harada@omu.ac.jp (A.H.); 2 Department of Applied Chemistry, Graduate School of Engineering, Osaka Metropolitan University, 1-1 Gakuen-cho, Naka-ku, Sakai 599-8531, Osaka, Japan

**Keywords:** liposome, particle size, pH-responsive, polysaccharide, cholesterol

## Abstract

The liposome particle size is an important parameter because it strongly affects content release from liposomes as a result of different bilayer curvatures and lipid packing. Earlier, we developed pH-responsive polysaccharide-derivative-modified liposomes that induced content release from the liposomes under weakly acidic conditions. However, the liposome used in previous studies size was adjusted to 100–200 nm. The liposome size effects on their pH-responsive properties were unclear. For this study, we controlled the polysaccharide-derivative-modified liposome size by extrusion through polycarbonate membranes having different pore sizes. The obtained liposomes exhibited different average diameters, in which the diameters mostly corresponded to the pore sizes of polycarbonate membranes used for extrusion. The amounts of polysaccharide derivatives per lipid were identical irrespective of the liposome size. Introduction of cholesterol within the liposomal lipid components suppressed the size increase in these liposomes for at least three weeks. These liposomes were stable at neutral pH, whereas the content release from liposomes was induced at weakly acidic pH. Smaller liposomes exhibited highly acidic pH-responsive content release compared with those from large liposomes. However, liposomes with 50 mol% cholesterol were not able to induce content release even under acidic conditions. These results suggest that control of the liposome size and cholesterol content is important for preparing stable liposomes at physiological conditions and for preparing highly pH-responsive liposomes for drug delivery applications.

## 1. Introduction

Stimulus-responsive nanomaterials have been widely studied for application in drug delivery systems [1]. Various types of stimuli such as temperature, pH, light, redox, and enzyme have been introduced to nanomaterials to improve their drug delivery performance regarding controlled drug release and time-spatial regulation of drug contents at the target site. Among these nanomaterials, several liposome-based nanomaterials have been approved already for clinical use [2,3]. The liposome particle size affects the migration behavior to lymph nodes, blood circulation time, induction capability of adaptive immune responses, and uptake by targeting cells [4,5,6,7]. Additionally, the amount of content leakage from lipid-based vesicles reportedly depends on the particle size [8,9,10,11]. In stimulus-responsive liposomes, Hossann et al. reported that thermosensitive liposomes, composed of 1,2-dipalmitoyl-*sn*-glycero-3-phosphocholine (DPPC)/1,2-distearoyl-*sn*-glycero-3-phosphocholine (DSPC)/1,2-dipalmitoyl-*sn*-glycero-3-phosphoglyceroglycerol (50/20/30, mol/mol/mol), increased the amount of carboxyfluorescein released from liposomes with decreasing liposome size in the size range of 68–190 nm. The results were attributable to an increase in the membrane curvature and looser packing of the phospholipids [12]. Although many reports have described the effect of size on leakage from liposomes, few investigations have examined size effects on content release from stimulus-responsive vesicles. It is likely that stimulus-responsive liposomes show different stability and release profiles depending on their size. Therefore, investigating the effects of their particle size on various functions is important.

To control the physical and chemical properties of phospholipid-based nanoparticles, particle surfaces have been modified with materials such as peptides, proteins, carbohydrates, polysaccharides, poly(ethylene glycol), and antibodies [13,14,15,16]. Among these materials, polysaccharides, naturally occurring polymers, show biocompatibility, biodegradability, and non-immunogenicity [16,17]. Moreover, compared with uncoated liposomes, polysaccharide-coated liposomes enhance the resistance to harsh gastrointestinal environments and reduce drug leakage from liposomes caused by the increasing biochemical stability [17,18]. As reported by Li et al., because chitosan formed an electrostatic protective layer, chitosan-coated phospholipid vesicles in the range of approximately 200 nm reduced the released amount of Atlantic salmon protein hydrolysates in simulated gastric fluid and simulated intestinal fluid compared with uncoated vesicles [19]. Bai et al. reported that carboxymethylchitosan coating of liposomes prevented the hydrolysis and protonation of phospholipids, which enhanced the stability of liposomes with 148 nm size and increased the release of coix seed oil from the liposomes compared with the uncoated one [20]. Consequently, coating of polysaccharide derivatives onto small lipid vesicles is an effective means of increasing the stability and changing the drug release profile.

In addition to surface modification of functional polymers, lipid composition is also an important factor affecting liposome stability. Lipid composition affects membrane rigidity and the interaction between hydrophobic acyl chains. Takechi-Haraya et al. reported that incorporation of cholesterol (Chol) in egg phosphatidylcholine (EPC) transformed liposome membranes into an ordered structure and that it increased the bending modulus of lipid membranes, indicating that EPC liposome membranes containing Chol are more rigid [21]. Farzaneh et al. reported that EPC/Chol/1,2-distearoyl-phosphatidylethanolamine-methyl-polyethyleneglycol conjugate (mPEG-DSPE) liposomes leaked contents more slowly than EPC/mPEG-DSPE liposomes [22]. These reports indicated that incorporating Chol into EPC liposomes enhances liposome stability. In addition, saturated phospholipid-containing liposome membranes, DPPC or DPPC/1,2-dioleoyl-3-trimethylammonium-propane (DOTAP) (90/10), showed high membrane rigidity compared with unsaturated phospholipid-containing lipid membranes, EPC or 1,2-dioleoyl-*sn*-glycero-3-phosphocholine/DOTAP (90/10) [21]. Farzaneh et al. reported that substitution of DPPC for EPC in phospholipid/Chol/mPEG2000-DSPE liposomes decreased content leakage from the liposomes [22]. Hąc-Wydro et al. reported the molecular area of 1,2-dioleoyl-*sn*-glycero-3-phosphocholine (unsaturated phospholipids) in a Langmuir monolayer as larger than that of DSPC (saturated phospholipids) [23]. Unsaturated phospholipids have bending acyl chains because of a double bond in their acyl chain, inducing looser packing in the liposome membrane and increasing the intermolecular distance. Saturated phospholipids make lipid membranes more stable than unsaturated phospholipids because saturated chains are packed more closely. Moreover, they allow stronger interaction between phospholipids. Incorporation of Chol and substitution of saturated phospholipids for unsaturated phospholipids affect liposome stability and the release behavior of liposomes. Consequently, investigating the lipid composition effects on the physicochemical properties of stimuli-responsive liposomes is important.

Responsiveness to pH, a typical internal stimulus, is particularly important for designing functional nanomaterials responding to intracellular acidic compartments (endo/lysosomes) and/or weakly acidic pH within the tumor microenvironment, which provides effective delivery approaches for drugs acting at intracellular spaces such as nucleic acids, proteins, or peptides. We have provided pH-responsive properties to liposomes by incorporating polysaccharide derivative possessing pH-responsive functional groups. 3-Methylglutarylated dextran, which was conjugated with dextran and 3-methylglutarylated (MGlu) groups, released contents from the liposomes under weakly acidic pH conditions [24]. An MGlu group includes a hydrophobic structure next to a carboxy group. Therefore, protonation of a carboxy group in an MGlu group makes the MGlu group more hydrophobic in endosomal weakly acidic pH, destabilizing liposome membranes or endosome membranes. To date, we have investigated the pH-responsive properties of various polysaccharides conjugated with MGlu groups (e.g., curdlan, Aquaβ, mannan, hyaluronic acid, or chondroitin sulfate) [24,25,26,27,28]. Among them, Aquaβ, a β1,3-β1,6-branched β-glucan derived from *Aureobasidium pullulans*, is particularly useful as a backbone of pH-responsive polysaccharide derivative because of the increased modification amount of Aquaβ derivative to liposomes through its branching structure [25]. In addition, 3-methylglutarylated Aquaβ (MGlu-Aquaβ) induced content release from liposomes under weakly acidic conditions. To date, we have adjusted the size of liposomes modified with pH-responsive polysaccharide derivatives from 100 to 200 nm because the size range is efficient for endocytosis by the cells [24,25,26,27,28]. However, it remains fundamentally unclear how the size of polysaccharide-derivative-modified liposomes affects their physicochemical properties.

For the present study, we investigated the physical properties of different-sized liposomes modified with MGlu-Aquaβ to identify the appropriate liposome size. We controlled the size of liposomes modified with MGlu-Aquaβ by extrusion through polycarbonate membranes of different pore sizes. Using extrusion, several passes through polycarbonate membranes allow for the reproducible formation of liposomes close to the membrane pore sizes [29]. Other methods for phospholipid-based particle size control, such as sonication and high-pressure homogenization, entail several shortcomings such as possible phospholipid degradation, low reproducibility, high size polydispersity for sonication, and high and variable size distributions for high-pressure homogenization. Microfluidic devices can adjust the lipid-based vesicle size by changing the flow rate ratio of the aqueous buffer to phospholipid-containing alcohol, thereby reproducibly controlling the size of lipid-based vesicles. However, microfluidic devices are mainly applicable for the control of lipid-based vesicle sizes in the nanoscale range of less than 200 nm [29,30]. Therefore, for this study, extrusion was selected as the method for controlling the liposome size in a wide size range. We evaluated the particle size effects of MGlu-Aquaβ-modified liposomes on pH-responsive properties and liposome stability.

## 2. Materials and Methods

### 2.1. Materials

β-1,3-1,6-Glucan (Aquaβ, Mw: 100 kDa, the degree of branching is 0.71 calculated from ^1^H NMR) was kindly donated by Osaka Soda Co., Ltd. (Osaka, Japan). Egg yolk phosphatidylcholine (EYPC, Figure S1) was kindly donated by NOF Corp. (Tokyo, Japan). Chol (Figure S1), DPPC (Figure S1), and *p*-xylene-bis-pyridinium bromide (DPX) were purchased from Sigma-Aldrich Corp. (St. Louis, MO, USA). Pyranine and Triton X-100 were obtained from Tokyo Chemical Industry Co., Ltd. (Tokyo, Japan). Phenol and phospholipid C test Wako were from Fujifilm Wako Pure Chemical Corp. (Osaka, Japan). Sulfuric acid, methanol, and chloroform were from Nacalai Tesque, Inc. (Kyoto, Japan). Sodium hydrogen carbonate was purchased from Kishida Chemical Co., Ltd. (Osaka, Japan). Cellulose tubing for dialysis (MWCO: 12,000–14,000) was obtained from Viskase Companies, Inc. (Lombard, IL, USA). MGlu-Aquaβ (MGlu unit: 56% and decylamide group-containing anchor unit: 4% per OH units in Aquaβ) was synthesized as described in an earlier report [25] (Scheme S1).

### 2.2. Preparation of Polysaccharide-Derivative-Modified Liposomes

For EYPC/Chol liposomes, a given amount (5–10 mg) of EYPC and Chol (EYPC/Chol = 100/0, 80/20, 50/50, mol/mol) dissolved in chloroform was added to a round-bottom flask. After evaporation of chloroform using a rotary evaporator, MGlu-Aquaβ (lipid:polysaccharide derivative = 7:3, wt:wt) dissolved in methanol was added to the flask. The solvent was evaporated. The remaining organic solvent was removed further under vacuum. The obtained mixed thin film of lipids and MGlu-Aquaβ was dispersed in phosphate-buffered saline (PBS) by a brief vortex. The liposome suspension was hydrated further by freeze–thaw cycles. The liposome suspension was centrifuged (6000 rpm, 10 min, 4 °C), and the pellet was resuspended in PBS. This procedure was performed three times to remove unmodified MGlu-Aquaβ molecules from the liposomes. The removal of free MGlu-Aquaβ was confirmed by measuring the amount of MGlu-Aquaβ in the third supernatants using a phenol–sulfuric acid method [31,32,33,34]. To control the liposome size, for EYPC/Chol liposomes, EYPC/Chol liposome suspension was extruded through polycarbonate membranes of 1000, 400, or 200 nm pore size at room temperature (EL-1000, EL-400, and EL-200). EL-1000 and EL-400 were further centrifuged (12,000 rpm, 30 min, 4 °C) twice to remove smaller liposomes. EL-200 was concentrated by centrifugation (55,000 rpm, 1 h, 4 °C).

For DPPC/Chol liposomes, DPPC and Chol (DPPC/Chol = 50/50, mol/mol) dissolved in chloroform were added to a round-bottom flask. After evaporation of chloroform using a rotary evaporator, MGlu-Aquaβ (lipid:polysaccharide derivative = 7:3, wt:wt) dissolved in methanol was added to the flask. The solvent was evaporated. The remaining organic solvent was further removed under vacuum. The obtained mixed thin film of lipids and MGlu-Aquaβ was dispersed in PBS by a brief vortex. The liposome suspension was centrifuged (6000 rpm, 10 min, 4 °C), and the pellet was resuspended in PBS. This procedure was performed three times to remove unmodified MGlu-Aquaβ molecules from the liposomes. DPPC/Chol liposome suspension was extruded through polycarbonate membranes of 1000, 400, or 50 nm pore size above 50 °C (DL-1000, DL-400, and DL-50). DL-1000 and DL-400 were further centrifuged (12,000 rpm, 30 min, 4 °C) twice to remove smaller liposomes. DL-50 was concentrated by centrifugation (55,000 rpm, 1 h, 4 °C).

### 2.3. Characterization of Polysaccharide-Derivative-Modified Liposomes

Lipid concentration in liposome suspensions was measured using a phospholipid C test Wako. The size distribution of the liposomes was measured using a Zetasizer Nano ZS (Malvern Instruments, Ltd., Worcestershire, UK). To evaluate liposome stability, liposomes (lipid concentration: 0.5 mM) were incubated at 37 °C for three weeks. The diameters were measured using a Zetasizer Nano ZS at given time points. The polysaccharide content per lipid was measured using a phenol–sulfuric acid method. Amounts of 200 µL of 5% phenol aqueous solution and 1 mL of 98% sulfuric acid were sequentially added to 200 μL aqueous solution of β-glucan-derivative-modified liposomes. The mixture was vortexed and then incubated for 1 h. For a calibration curve, mixtures of 0–250 µg/mL of MGlu-Aquaβ and lipid suspension at an equal concentration to tested MGlu-Aquaβ-modified liposomes were prepared. Absorption spectra (450–550 nm) for samples were measured, and second-derivative spectra were used for the calculation of polysaccharide contents to eliminate the residual background signal effects based on lipid suspension. Data were obtained as an average of at least three measurements of different samples prepared from different batches.

### 2.4. pH-Responsive Properties of Polysaccharide-Derivative-Modified Liposomes

Pyranine was selected as a model dye to evaluate the pH-responsive properties of the liposomes. Pyranine fluorescence is quenched by DPX inside of the liposomes, but this molecule shows intense fluorescence when released from the liposome [35], which allows easy monitoring of pH-responsive content release from the liposomes. Pyranine-loaded liposomes were prepared as described above except that the mixed thin film of lipids and polysaccharide derivatives was dispersed in aqueous 35 mM pyranine, 50 mM DPX, and 25 mM phosphate solution (pH 7.4). Liposomes encapsulating pyranine (lipid concentration: 20 μM) were added to PBS (pH 7.4), or the solution was adjusted to weakly acidic pH buffered with acetate buffer at 37 °C in a quartz cuvette (total volume: 2.5 mL). Fluorescence intensity at 512 nm of the mixed suspension was followed with excitation at 416 nm using a spectrofluorometer (FP-6500; Jasco Corp., Tokyo, Japan). The release percentage of pyranine from liposomes was defined as

Release (%) = (*F*_t_ − *F*_i_)/(*F*_f_ − *F*_i_) × 100,

where *F*_i_ and *F*_t_ respectively denote the initial fluorescence intensity and the intermediary fluorescence intensity of the liposome suspensions. *F*_f_ is the fluorescent intensity of the liposome suspension after the addition of a typical nonionic surfactant, Triton X-100 (final concentration: 0.1%, which is much higher than its critical micellar concentration, leading the complete destabilization of the lipid bilayer structure). Data were obtained as an average of at least three measurements of different samples prepared from different batches.

## 3. Results and Discussion

### 3.1. Preparation of Size-Controlled Polysaccharide-Derivative-Modified Liposomes

**Scheme 1:**
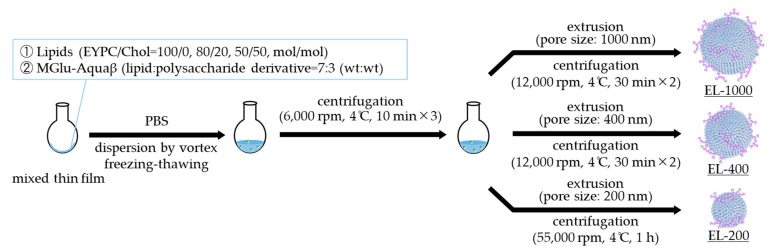
Preparation of size-controlled EYPC/Chol liposomes modified with polysaccharide derivatives.

**Figure 1:**
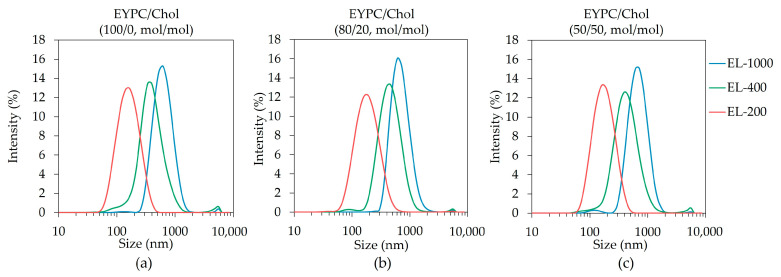
Size distribution of EYPC/Chol (100/0 (**a**), 80/20 (**b**), 50/50 (**c**), mol/mol) liposomes modified with polysaccharide derivative. Liposome size control was performed by extrusion with 1000, 400, or 200 nm polycarbonate membranes and subsequent centrifugation.

**Figure 2:**
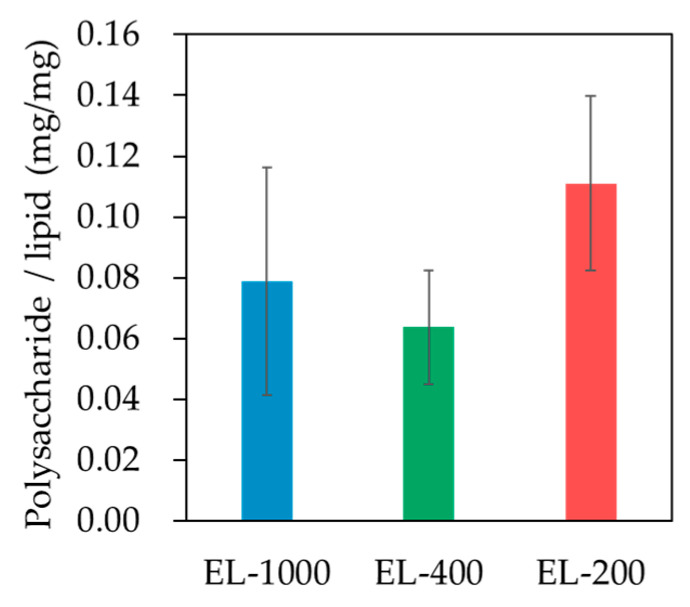
Polysaccharide derivative weight on EYPC/Chol (80/20, mol/mol) liposomes detected using phenol–sulfuric acid method. Liposomes were prepared by extrusion through 1000, 400, and 200 nm of polycarbonate membrane at lipid:polysaccharide derivative = 7:3 (wt:wt).

Size control of liposomes modified with MGlu-Aquaβ was performed by extrusion with polycarbonate membranes having various pore sizes (Scheme 1) based on a report of a study by Matsuoka et al. [36]. In this report, a liposome of diameter > 600 nm induced cell-based immune responses, whereas a liposome of diameter < 400 nm activated immune cells [36]. In addition, the size limit for clathrin-mediated endocytosis is regarded as less than 200 nm. Considering these reports, the sizes of 1000, 400, and 200 nm were chosen to evaluate the size impact for the properties of the liposomes. A mixed thin film composed of polysaccharide derivatives and lipids (EYPC/Chol = 80/20, mol/mol) was dispersed in PBS to produce the liposome suspension. After freeze–thaw processing, the centrifugation–resuspension cycle of the liposome suspensions was repeated three times to remove the free polysaccharide derivative, which was not modified onto liposomes. For liposome size control, the liposomes were passed through polycarbonate membranes of 1000, 400, or 200 nm pore size. Just after extrusion with 1000 and 400 nm membranes, liposomes possessed a wide size distribution (Figure S2). Therefore, the liposome suspensions were centrifuged further to remove smaller liposomes. The purified liposomes extruded with a polycarbonate membrane of 1000, 400, or 200 nm pore size (EL-1000, EL-400, or EL-200) showed 655.8 ± 31.2, 402.8 ± 5.8, or 162.2 ± 6.6 nm, respectively corresponding to the pore sizes of membranes (Table 1, Figure 1 and Figure S3). Polydispersity indexes (PDIs) of liposomes were 0.226, 0.248, and 0.185 for EL-1000, EL-400, and EL-200, respectively. Liposome morphology observed by a transmission electron microscopy (TEM) implied that liposomes were of spherical shape, and their sizes corresponding to the pore size of membranes were controlled by extrusion (Figure S4). The amounts of polysaccharide derivatives in each liposome were ascertained using the phenol–sulfuric acid method. The weight ratios of polysaccharide per lipid in EL-1000, EL-400, and EL-200 were 0.079 ± 0.037, 0.064 ± 0.019, and 0.111 ± 0.029 mg/mg, respectively (Figure 2). The modification amounts of polysaccharide derivatives on liposomes were almost identical irrespective of the pore size of the polycarbonate membrane used for extrusion. Polysaccharide-derivative-modified liposomes with all sizes possessed negative zeta potentials because of carboxy groups with polysaccharide derivatives modified on the liposomes (Table 1). The zeta potential for unmodified liposomes was −7.9 mV [25], which is less value compared with polysaccharide-derivative-incorporated liposomes. These data suggest that the liposome surface is modified with polysaccharide derivatives.

### 3.2. Cholesterol Content Effects on Liposome Stability

Effects of cholesterol contents in liposomes on the colloidal stability of EYPC liposomes were investigated, where liposomes were composed of EYPC and Chol at ratios of 100/0, 80/20, or 50/50 (mol/mol) (Scheme 1). Liposome particle sizes of EYPC/Chol (100/0 and 50/50, mol/mol) were also controlled by extrusion with 1000, 400, or 200 nm polycarbonate membranes. Average diameters of EYPC liposomes containing Chol were greater than those of EYPC/Chol (100/0, mol/mol) liposomes (Table 1). Lie et al. reported that increased Chol contents in the liposome membrane increased the particle size because the incorporation of Chol reconstructed the phosphatidylcholine (PC) bilayer into a more rigid and ordered structure, where PC/Chol liposomes contained vitamin E with different Chol contents (0, 33, and 50 mol%) [37]. Zhao et al. also reported that incorporating sterols into soy lecithin liposomes increased interaction with phospholipid acyl chains and strengthened the packing of bilayers, producing large liposomes [38]. In addition, Doskocz et al. reported that although the extrusion of 140 nm DPPC liposomes in liquid crystalline phase with polycarbonate membranes of 50 nm pore size reduced the particle size to 80 nm, 140 nm DPPC liposomes extruded in the solid-ordered phase did not change the size [39]. The authors stated that this was attributed to the high bending modulus of liposome membranes in the solid-ordered phase [39]. Incorporating Chol in EYPC liposomes enhanced the bending modulus [21]. Thus, EYPC liposomes with Chol might become a higher bending modulus of membranes and retain a larger particle size after extrusion than those without Chol.

**Figure 3:**
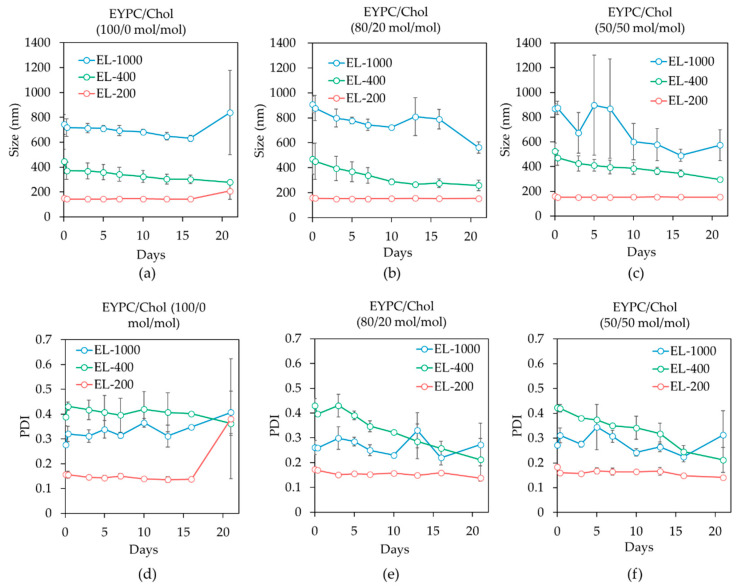
(**a**–**c**) Particle size and (**d**–**f**) PDI of EYPC/Chol (100/0, 80/20, 50/50, mol/mol) liposomes modified with polysaccharide derivative. Liposome size control was performed by extrusion with 1000, 400, or 200 nm polycarbonate membranes.

The colloidal stability of EYPC/Chol (100/0, 80/20, 50/50, mol/mol) liposomes of different sizes was investigated by measuring the particle size of liposomes incubated at 37 °C for different durations. The average particle sizes were maintained in two weeks for all EYPC liposomes (Figure 3a–c). This lack of a change in size might be attributed to electrostatic repulsion between negatively charged polysaccharide derivatives modified on the liposomes, which consequently improved the colloidal stability of liposomes. For EYPC/Chol (50/50, mol/mol) liposomes, EL-1000 showed some unstable behavior during the first week of incubation. Although electrorepulsion by polycarboxylates suppresses the aggregation of liposomes during liposome incubation, EL-1000 has relatively large volume compared with EL-400 and EL-200, which might increase the possibility of collision between liposomes especially during the first week, resulting in an increase in standard deviation. After 10 days, electrorepulsion by polycarboxylates might unravel such aggregation of EL-1000, leading to the gradual size decrease. Müller et al. reported that electrostatic repulsion of particles with a large absolute zeta potential value prevented particle aggregation, leading to a stable colloidal suspension [40]. Zhou et al. reported that pectin-coated liposomes showed improved stability and drug leakage percentages of liposomes compared with uncoated liposomes because of the increased density of the negative charge [41]. However, the PDI of EL-200 prepared with 0 mol% Chol was drastically increased after three weeks of incubation (Figure 3d). This increase might derive from the detachment of polysaccharide derivatives from liposomes and induction liposome destabilization. Smistad et al. reported that soybean phosphatidylcholine liposomes modified with octyl chain-conjugated hydroxyethyl cellulose (HEC) showed an increased particle size 24 weeks after incubation at 4 °C, whereas liposomes modified with hexadecyl chain-conjugated HEC were stable for 24 weeks [42]. This stability resulted from the weak interaction between short alkyl chains and liposome membranes compared with long alkyl chain groups. EL-200 might possess high membrane curvature and low lipid packing. Therefore, the liposome membranes of EL-200 might weakly interact with decyl chains in polysaccharide derivatives. By contrast, the colloidal stability of EL-200 was clearly improved by introducing Chol: the particle size of EL-200 had retained similar values after three weeks (Figure 3e,f). Lie et al. reported that EPC liposomes containing Chol suppressed the particle size increase as time progressed compared with EPC liposomes [36]. In addition, Serfis et al. reported in a mixed Langmuir monolayer of EPC and Chol that molecular areas decreased compared with ideal molecular areas calculated from a pure EPC and Chol monolayer using the additive rule, indicating that incorporating Chol to an EPC monolayer induced condensation and more tight packing of EPC and Chol within a lipid monolayer [43]. As already reported by Bhattacharya et al., for ^1^H NMR analysis, the increase in Chol in phospholipid vesicles induced the broadening of the proton signal of methylene units in acyl chains and restricted the relaxation time of the methylene, indicating immobilization of methylene segments [44]. This immobilization results in increased interaction between lipid acyl chains and the formation of tight packing in membranes [44]. Incorporation of Chol into polysaccharide-derivative-modified liposomes might increase the interaction between acyl chains in EYPC and form tighter packing, resulting in increased stabilization of EYPC liposomes with Chol compared with those without Chol. These results implied that incorporating 20 mol% Chol to liposomes was sufficient to stabilize polysaccharide-derivative-modified liposomes.

### 3.3. Liposome Size Effects on pH-Responsive Properties

**Figure 4:**
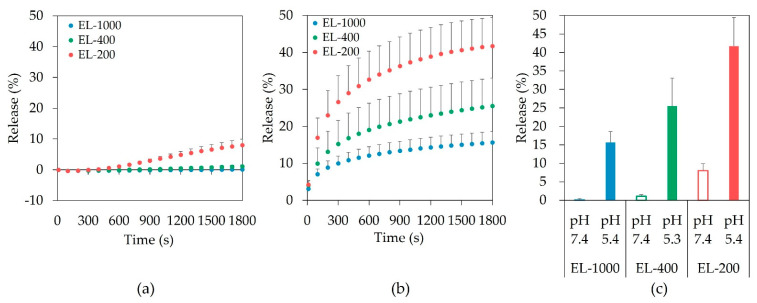
Pyranine release from various size EYPC/Chol (80/20, mol/mol) liposomes modified with polysaccharide derivative in weakly acidic conditions. Time courses of pyranine release at (**a**) pH 7.4 and (**b**) pH 5.4 or 5.3, and (**c**) release percentages in 30 min of pyranine liposomes.

To evaluate liposome size effects on the pH-responsive property of polysaccharide-derivative-modified liposomes, the release profile from pyranine-encapsulating EYPC/Chol (80/20, mol/mol) liposomes was investigated. Liposomes encapsulating pyranine and DPX as a fluorescent dye and a quencher, respectively, were prepared using the same method as that described above. Fluorescence derived from pyranine is quenched by DPX in liposomes, but the fluorescence intensity was drastically increased when these molecules were released from liposomes because of the decreasing quenching effect. Pyranine fluorescence intensity after the addition of Triton X-100 to a given amount of liposome suspension was almost identical, indicating that the pyranine molecule content per liposome is almost the same in evaluated liposomes (Figure S5). Yuba et al. reported that the p*K*a of 3-methylglutarylated polysaccharides (e.g., MGlu-curdlan) was in the range of 5.7–6.0, and MGlu-curdlan formed hydrophobic domains in weakly acidic pH, which induces the destabilization of the liposome membranes via hydrophobic interaction, leading to content release from 3-methylglutarylated polysaccharide-modified EYPC liposomes [26]. Pyranine was released only slightly from pyranine-loaded EL-1000, EL-400, and EL-200 at pH 7.4 (Figure 4a). Pyranine release occurred with all size-controlled liposomes at pH 5.4, which corresponds to the pH within endosomes. Moreover, small liposomes tended to show higher release capability than large liposomes (Figure 4b,c). In addition, the initial release rate of small liposomes was higher than that of large liposomes (Figure S6). This high rate resulted from the high curvature of the lipid membrane in small liposomes. Small liposomes have higher curvature of lipid membranes than large liposomes; that higher curvature is unstable because of the looser packing of the membrane [8,11]. Hatzakis et al. reported that small liposomes increased the density of packing defects compared with large liposomes [45]. Bending a bilayer created mismatches to the packing of phospholipids, and hydrophobic moieties were exposed to an aqueous medium. EL-200 leaked pyranine slightly under neutral conditions compared with EL-1000 and EL-400, which might be caused by increased packing defects in small liposomes and decreased liposome stability. In addition, hydrophobic domains formed by polysaccharide derivatives at weakly acidic pH might be prone to access hydrophobic moieties of phospholipids because small liposomes possess more packing defects and expose hydrophobic moieties of phospholipids. Therefore, the liposomal membrane of small liposomes might be destabilized by polysaccharide derivatives more easily than those of large liposomes. The effects of the Chol content on the release from liposomes were evaluated using EYPC/Chol (80/20, 50/50, mol/mol) liposomes extruded with a 400 nm membrane. Results show that EYPC/Chol (50/50, mol/mol) liposomes had a lower release because of their rigid membrane compared with the release of EYPC/Chol (80/20, mol/mol) liposomes (Figure S7). These results suggest that EYPC/Chol (80/20, mol/mol) liposomes were more suitable lipid components for pH-responsive liposomes than liposomes with the other Chol ratios to balance their stability and pH-responsive drug release capabilities.

### 3.4. Lipid Composition Effects

EYPC/Chol (50/50, mol/mol) liposomes showed decreased release performance compared with EYPC/Chol (20/20, mol/mol) liposomes (Figure S7). Substitution of saturated phospholipids for EYPC is assumed to decrease the content release performance from liposomes further because saturated phospholipids form more rigid membranes. However, lipid composition composed of saturated phospholipids provides several benefits for liposome properties in addition to liposome stability, such as drug encapsulation efficiency and cellular association [21,22,37,38]. Therefore, we evaluated pH-responsive properties of liposomes composed of DPPC, saturated phospholipid. Polysaccharide-derivative-modified liposomes comprising DPPC and Chol (50/50, mol/mol) were prepared with extrusion through polycarbonate membranes of 1000, 400, or 50 nm pore size (DL-1000, DL-400, or DL-50). DPPC liposome membranes under 42 °C, which is its phase transition temperature, take a gel phase, which is a solid-ordered phase and forms a compact packing with minimal mobility. However, 50 mol% Chol-incorporated DPPC liposome membranes are in a liquid-ordered phase at room temperature; moreover, they possess lower rigidity than pure DPPC liposome membranes [21,46]. Bhattacharya et al. reported that the incorporation of Chol to DPPC membranes set DPPC molecules apart and reduced the interaction between DPPC molecules, inducing lower rigidity of DPPC membranes containing Chol [44]. Because DPPC/Chol (50/50, mol/mol) liposomes were more rigid than EYPC/Chol (50/50, mol/mol) liposomes [21], DPPC/Chol (50/50, mol/mol) liposomes are presumed to possess high stability. DPPC/Chol (50/50, mol/mol) liposome membranes may be looser packing, increasing content release compared with pure DPPC liposomes. We selected the lipid composition of DPPC and Chol at a ratio of 50:50 as the lipid composition, which contains saturated phospholipids and which is susceptible to induce liposome membrane destabilization by insertion of hydrophobized polysaccharide derivatives under weakly acidic conditions. The particle sizes of DL-1000, DL-400, and DL-50 were 1019.4 ± 137.2, 560.2 ± 39.9, and 104.6 ± 3.3 nm, respectively corresponding to the pore sizes of polycarbonate membranes used for extrusion (Table 2 and Figure S8).

**Figure 5:**
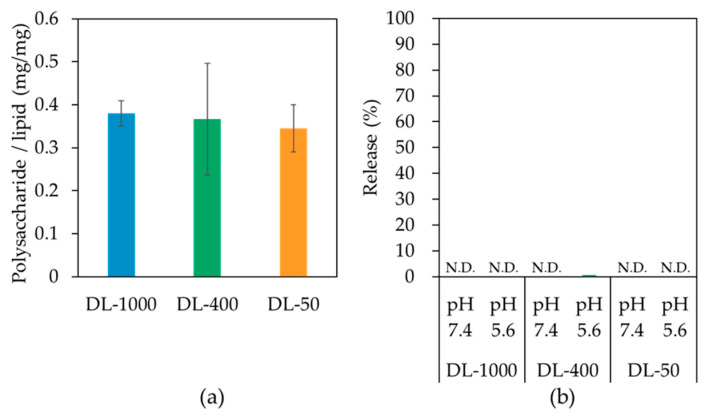
(**a**) Polysaccharide derivative weight on DPPC/Chol (50/50, mol/mol) liposomes detected using phenol–sulfuric acid method. Liposomes were prepared by extrusion through 1000, 400, and 50 nm of polycarbonate membrane at lipid:polysaccharide derivative = 7:3 (wt:wt). (**b**) Pyranine release from various size DPPC/Chol (50/50, mol/mol) liposomes modified with polysaccharide derivatives in neutral and weakly acidic conditions. Release percentages in 30 min of pyranine liposomes were calculated from fluorescein intensities after 30 min incubation and addition of Triton X-100.

Similarly to EYPC/Chol liposomes, the amounts of polysaccharide derivatives in each DPPC/Chol liposome were ascertained using the phenol–sulfuric acid method. The weights of polysaccharide derivatives per lipid in DL-1000, DL-400, and DL-50 were 0.380 ± 0.029, 0.366 ± 0.130, and 0.345 ± 0.055 mg/mg, respectively (Figure 5a). These amounts were three-fold to six-fold the amounts of EYPC/Chol liposomes. Hąc-Wydro et al. reported the packing degree for the mixture of stearic acid (saturated fatty acid) and 1,2-dioleoyl-*sn*-glycero-3-phosphocholine (unsaturated phospholipid) as lower than that of stearic acid and DSPC (saturated phospholipid), which indicates strong interaction between the hydrophobic chains of saturated fatty acid and saturated phospholipid [23]. Therefore, saturated decyl chains conjugated with polysaccharide derivatives might interact more strongly with DPPC, saturated phospholipid, than EYPC, unsaturated phospholipid, within each liposome membrane. Next, the pH-responsive release behavior of DPPC/Chol liposomes modified with polysaccharide derivatives was evaluated using liposomes encapsulating pyranine and DPX. Irrespective of the liposome size, none of the liposomes released the pyranine both under neutral (pH 7.4) and weakly acidic pH (pH 5.6) conditions (Figure 5b). Takechi-Haraya et al. reported that DPPC/Chol (50/50, mol/mol) liposomes showed a higher bending modulus than EYPC/Chol (50/50, mol/mol) liposomes, indicating that DPPC/Chol liposomes possess high rigid lipid membranes [21]. Gürsoy et al. reported that DPPC/Chol (2:1, mol/mol) liposomes leaked less amounts of content than EYPC/Chol (2:1, mol/mol) liposomes [47]. Also, Jo et al. reported that the degree of release from EYPC liposomes modified with pH-responsive polymer, poly(*N*-isopropylacrylamide-*co*-methacrylic acid-*co*-octadecylacrylate) conjugated to glucose oxidase, was higher than that from DPPC liposomes modified with the polymer under the weakly acidic conditions [48]. These reports were attributable to the more rigid DPPC liposome membranes than EYPC liposome membranes. The driving force for liposome membrane destabilization of polysaccharide derivatives was hydrophobic domains formed by MGlu groups in weakly acidic pH [26]. In addition, polysaccharide derivatives, which showed high hydrophobicity in the high pH region, induced increased content release in the high pH region [26]. The hydrophobic domains formed by MGlu groups in polysaccharide derivatives might not form enough hydrophobic domains to destabilize rigid DPPC/Chol (50/50, mol/mol) membranes. Consequently, the high rigidity of DPPC/Chol (50/50, mol/mol) liposomes might prevent protonated polysaccharide derivatives from destabilizing liposome membranes, resulting in a lack of pyranine release from liposomes. Therefore, our results suggest that EYPC/Chol liposomes are a more suitable lipid component for pH-responsive liposomes than DPPC/Chol liposomes.

## 4. Conclusions

Size control of polysaccharide-derivative-modified liposomes was achieved by extrusion. The polysaccharide derivative contents per lipid did not change irrespective of the liposome size. Incorporation of cholesterol into liposomes enhanced the colloidal stability of liposomes, whereas high contents of cholesterol in liposomes suppressed the release behavior from liposomes responding to weakly acidic pH. Liposome membranes composed of DPPC and Chol increased the modification amounts of polysaccharide derivatives, but no release from DPPC/Chol liposomes was detected. Therefore, it is important that pH-responsive liposomes modified with polysaccharide derivatives include the appropriate Chol ratio and not DPPC but EYPC in liposomes. Small liposomes modified with pH-responsive polysaccharide derivatives induced higher content release than that of large liposomes in weakly acidic pH. These results support that the polysaccharide-derivative-modified liposome size adjusted from 100 nm to 200 nm in earlier reports was appropriate for exerting pH-responsive capabilities.

## Figures and Tables

**Table 1:** Characteristics of EYPC/Chol (100/0, 80/20, 50/50, mol/mol) Liposomes Modified with Polysaccharide Derivatives.

Liposome	Pore Size of Membrane for Extrusion	EYPC/Chol (100/0, mol/mol)			EYPC/Chol (80/20, mol/mol)			EYPC/Chol (50/50, mol/mol)		
		Size (nm)	PDI	ξ-Potential (mV)	Size (nm)	PDI	ξ-Potential (mV)	Size (nm)	PDI	ξ-Potential (mV)
EL-1000	1000 nm	588.5 ± 9.5	0.284	−31.7	655.8 ± 31.2	0.226	−31.3	630.1 ± 18.6	0.278	−30.1
EL-400	400 nm	346.4 ± 25.6	0.237	−29.6	402.8 ± 5.8	0.248	−16.1	384.5 ± 11.1	0.296	−24.8
EL-200	200 nm	142.6 ± 5.6	0.168	−29.3	162.2 ± 6.6	0.185	−15.0	155.5 ± 4.1	0.169	−36.0

**Table 2:** Sizes and PDIs of DPPC/Chol (50/50, mol/mol) Liposomes Modified with Polysaccharide Derivatives.

Liposome	Pore Size of Membrane for Extrusion	Size (nm)	PDI
DL-1000	1000 nm	1019.4 ± 137.2	0.298
DL-400	400 nm	560.2 ± 39.9	0.243
DL-50	50 nm	104.6 ± 3.3	0.181

## Data Availability

All data needed to evaluate the conclusions in the paper are present in the paper and/or the Supplementary Materials. Additional data available from authors upon reasonable request.

## Supplementary Materials

The following supporting information can be downloaded at: https://www.mdpi.com/article/10.3390/life13112158/s1, Scheme S1: Synthetic route for β-glucan derivatives; Figure S1: Structures of egg yolk phosphatidylcholine (EYPC), 1,2-dipalmitoyl-*sn*-glycero-3-phosphocholine (DPPC) and cholesterol (Chol); Figure S2: Size distribution of polysaccharide-derivative-modified liposomes with or without centrifugation after extrusion; Figure S3: Size and PDI of EYPC/Chol (100/0, 80/20, 50/50, mol/mol) liposomes modified with polysaccharide derivatives; Figure S4: TEM analysis of EL-1000, EL-400, and EL-200; Figure S5: Pyranine fluorescence per a given amount of liposome; Figure S6: Initial release rate of pyranine from various size liposomes in weakly acidic conditions; Figure S7: Time courses of pyranine release from polysaccharide-derivative-modified liposomes at pH 5.4; Figure S8: Size distribution of DPPC/Chol (50/50, mol/mol) liposomes modified with polysaccharide derivative.
