# Supplementary material for: Preparing Size-Controlled Liposomes Modified with Polysaccharide Derivatives for pH-Responsive Drug Delivery Applications
Source: Life (Basel). 2023 Nov 3;13(11):2158. doi: 10.3390/life13112158 (PMC10672248; doi:10.3390/life13112158)
Supplement: Supplementary file 1 [file life-13-02158-s001.zip › life-2629605-supplementary.pdf]

Supplementary materials

Preparing Size-Controlled Liposomes Modified with Polysaccharide Derivatives for pH-Responsive Drug Delivery Applications

Shin Yanagihara [1], Yukiya Kitayama [1,2], Eiji Yuba [1,2,*] and Atsushi Harada [1,2]

[1] Department of Applied Chemistry, Graduate School of Engineering, Osaka Prefecture University, 1-1 Gakuen-cho, Naka-ku, Sakai 599-8531, Osaka, Japan; liushin.ynghr@gmail.com (S.Y.); kitayama@omu.ac.jp (Y.K.); atsushi_harada@omu.ac.jp (A.H.)

[2] Department of Applied Chemistry, Graduate School of Engineering, Osaka Metropolitan University, 1-1 Gakuen-cho, Naka-ku, Sakai 599-8531, Osaka, Japan

* Correspondence: yuba@omu.ac.jp; Tel.: +81-72-247-6016

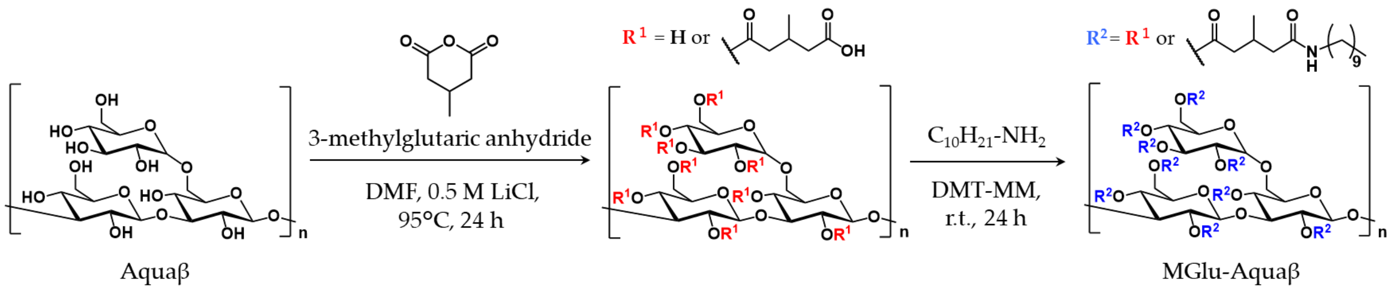

Scheme S1. Synthetic route for MGlu-Aquaβ having carboxy groups and alkyl chains as anchor units to liposomal membrane.

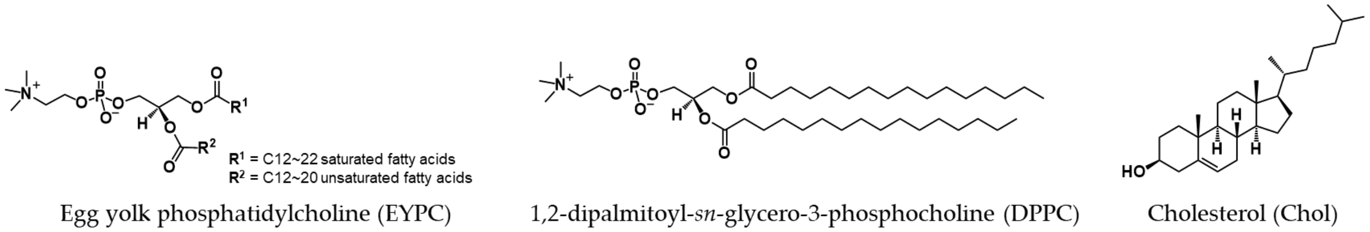

Figure S1. Structures of egg yolk phosphatidylcholine (EYPC), 1,2-dipalmitoyl-*sn*-glycero-3-phosphocholine (DPPC) and cholesterol (Chol).

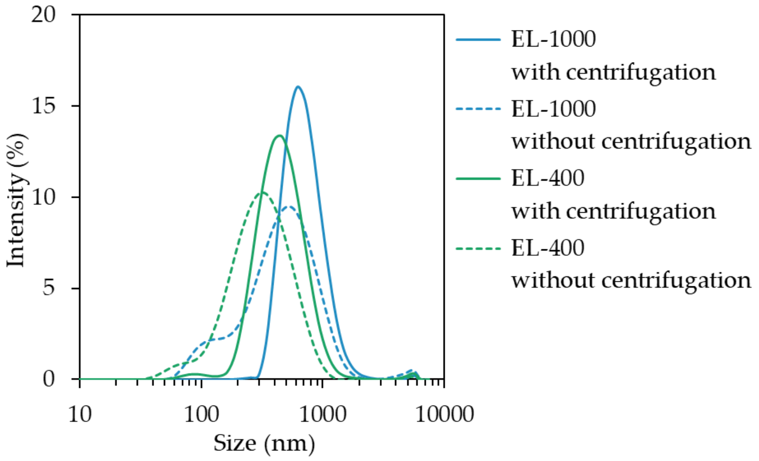

Figure S2. Size distribution of polysaccharide derivative-modified EYPC/Chol (80/20, mol/mol) liposomes with (solid lines) or without (dashed lines) centrifugation after extrusion. Liposome size control was performed by extrusion with 1,000 or 400 nm polycarbonate membranes (EL-1000 or EL-400).

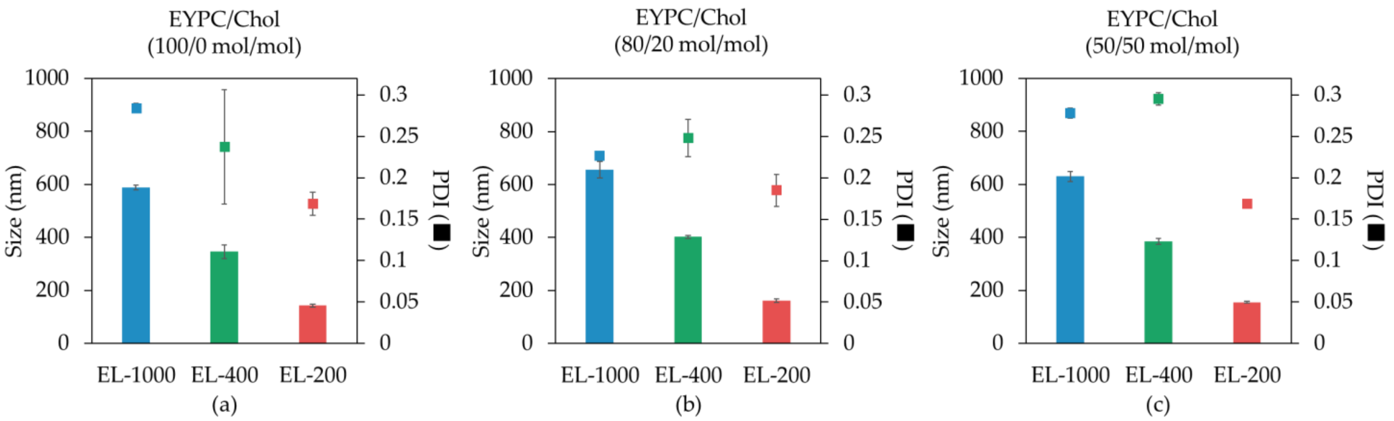

Figure S3. Size (bar) and PDI (square plot) of EYPC/Chol (100/0 (a), 80/20 (b), 50/50 (c), mol/mol) liposomes modified with polysaccharide derivatives. Liposome size control was performed by extrusion with 1,000, 400 or 200 nm polycarbonate membranes and subsequent centrifugation.

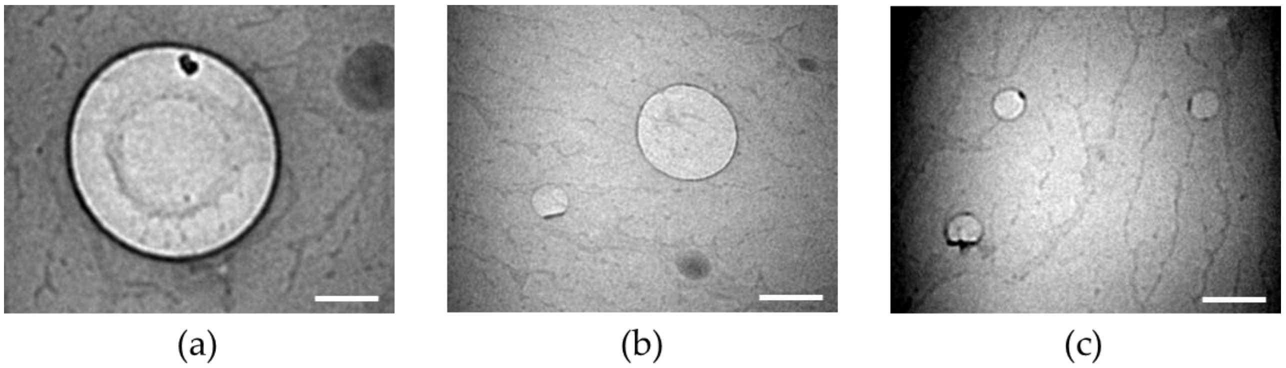

Figure S4. Transmission electron microscopic (TEM) analysis of (a) EL-1000, (b) EL-400, and (c) EL-200. Liposomes were dropped on a copper grid, and then negatively stained using phosphotungstic acid solution. Scale bars represent 200 nm.

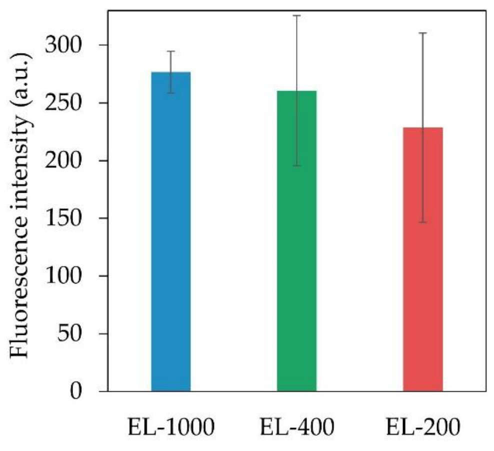

Figure S5. Fluorescence intensity of pyranine per a given amounts of lipids. After pyranine released from liposomes was tracked by measuring fluorescence intensity for 30 min, 10% Triton X-100 were added to liposomes and all pyranines encapsulated in liposomes were released.

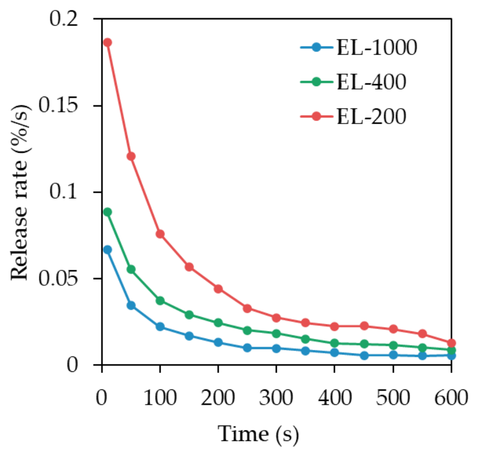

Figure S6. Initial release rate of pyranine from various size EYPC/Chol (80/20 mol/mol) liposomes modified with polysaccharide derivatives in weakly acidic conditions. Initial release rate was calculated from Figure 4b.

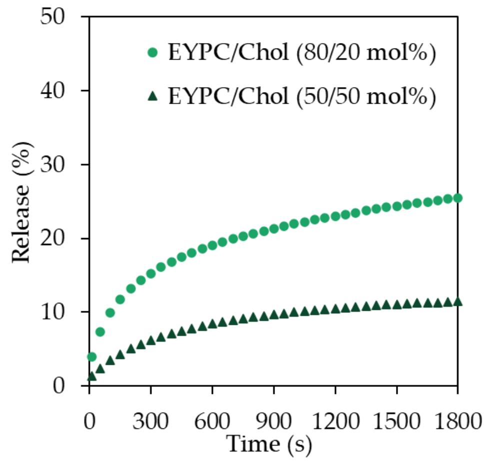

Figure S7. Time-courses of pyranine release from polysaccharide derivative-modified liposomes composed of 80/20 (circles) or 50/50 (triangles) mol/mol EYPC/Chol at pH 5.4. Pyranine-loaded liposomes were prepared with extrusion through 400 nm polycarbonate membrane.

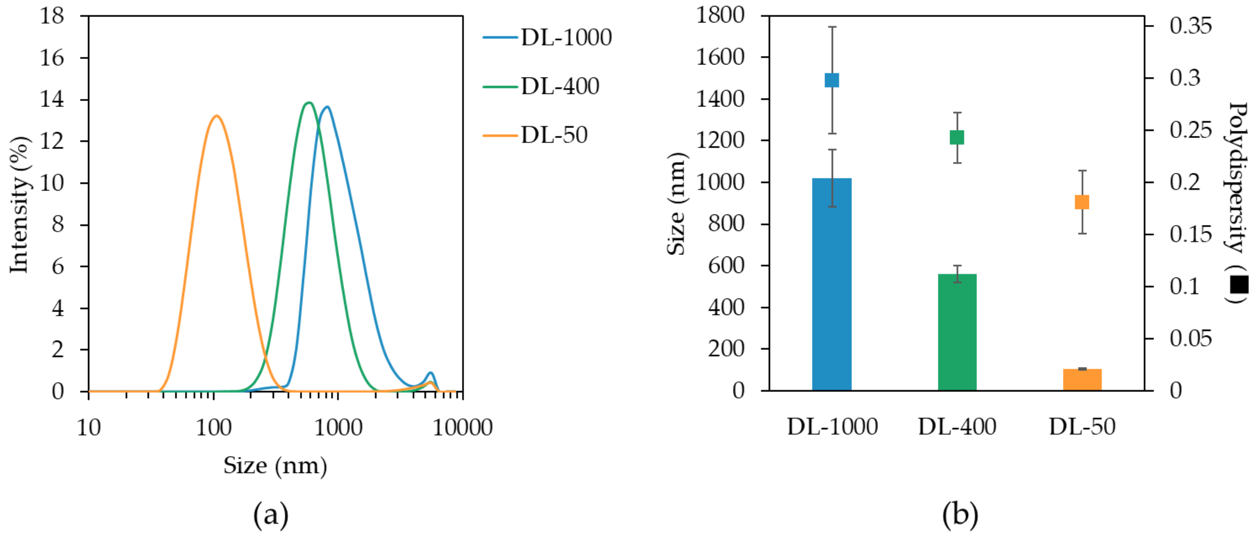

Figure S8. (a) Size distribution, and (b) Size (bar) and PDI (square plot) of DPPC/Chol (50/50, mol/mol) liposomes modified with polysaccharide derivatives. Liposome size control was performed by extrusion with 1,000, 400 or 50 nm polycarbonate membranes and subsequent centrifugation.
